# Supplementary material for: Ecology and Machine Learning-Based Classification Models of Gut Microbiota and Inflammatory Markers May Evaluate the Effects of Probiotic Supplementation in Patients Recently Recovered from COVID-19
Source: Int J Mol Sci. 2023 Apr 1;24(7):6623. doi: 10.3390/ijms24076623 (PMC10094838; doi:10.3390/ijms24076623)
Supplement: Supplementary file 1 [file ijms-24-06623-s001.zip › Supplementary Figures S1-S7.pptx]

## Slide 1
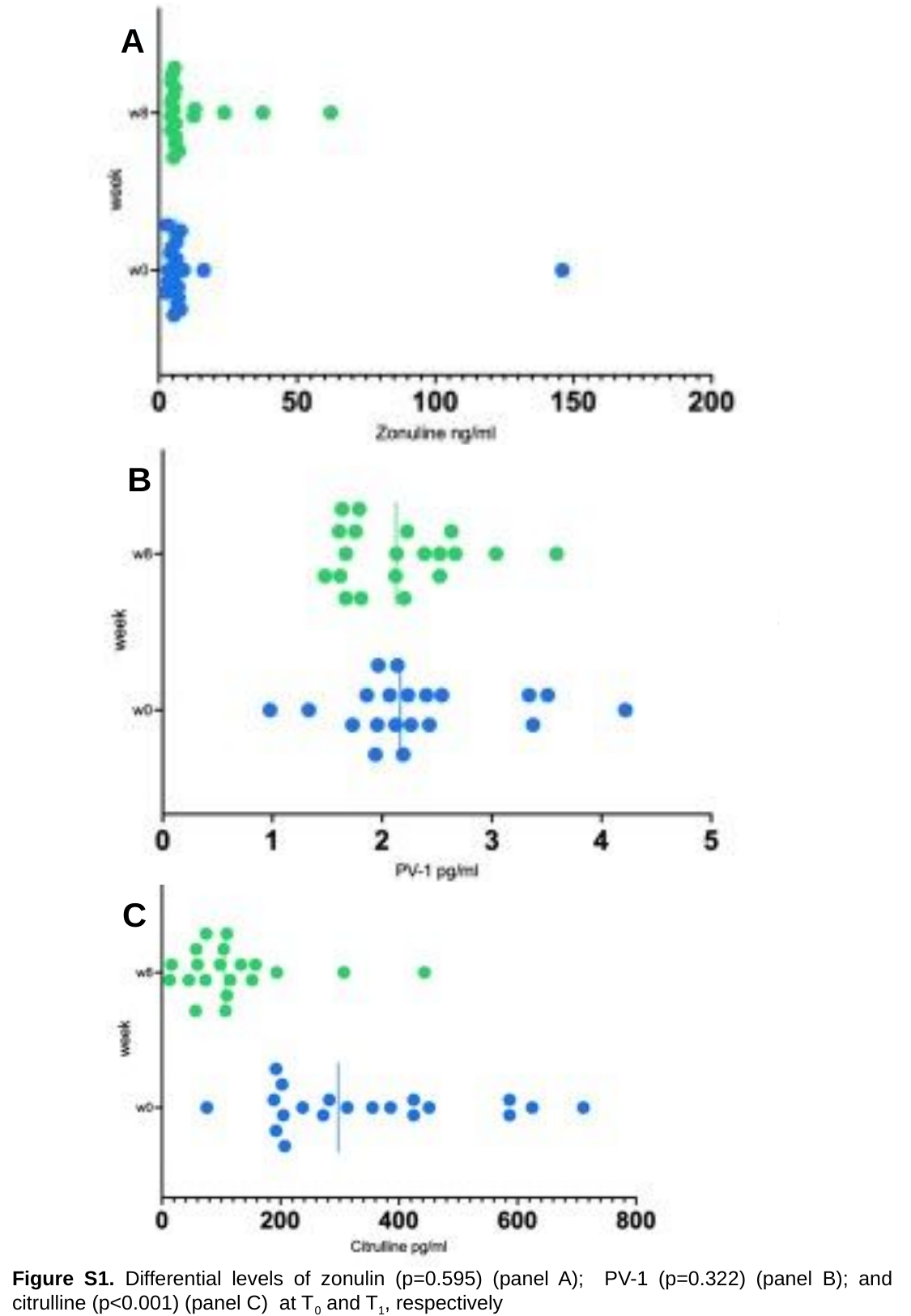

A
B
C
Figure S1. Differential levels of zonulin (p=0.595) (panel A); PV-1 (p=0.322) (panel B); and citrulline (p<0.001) (panel C) at T0 and T1, respectively

## Slide 2
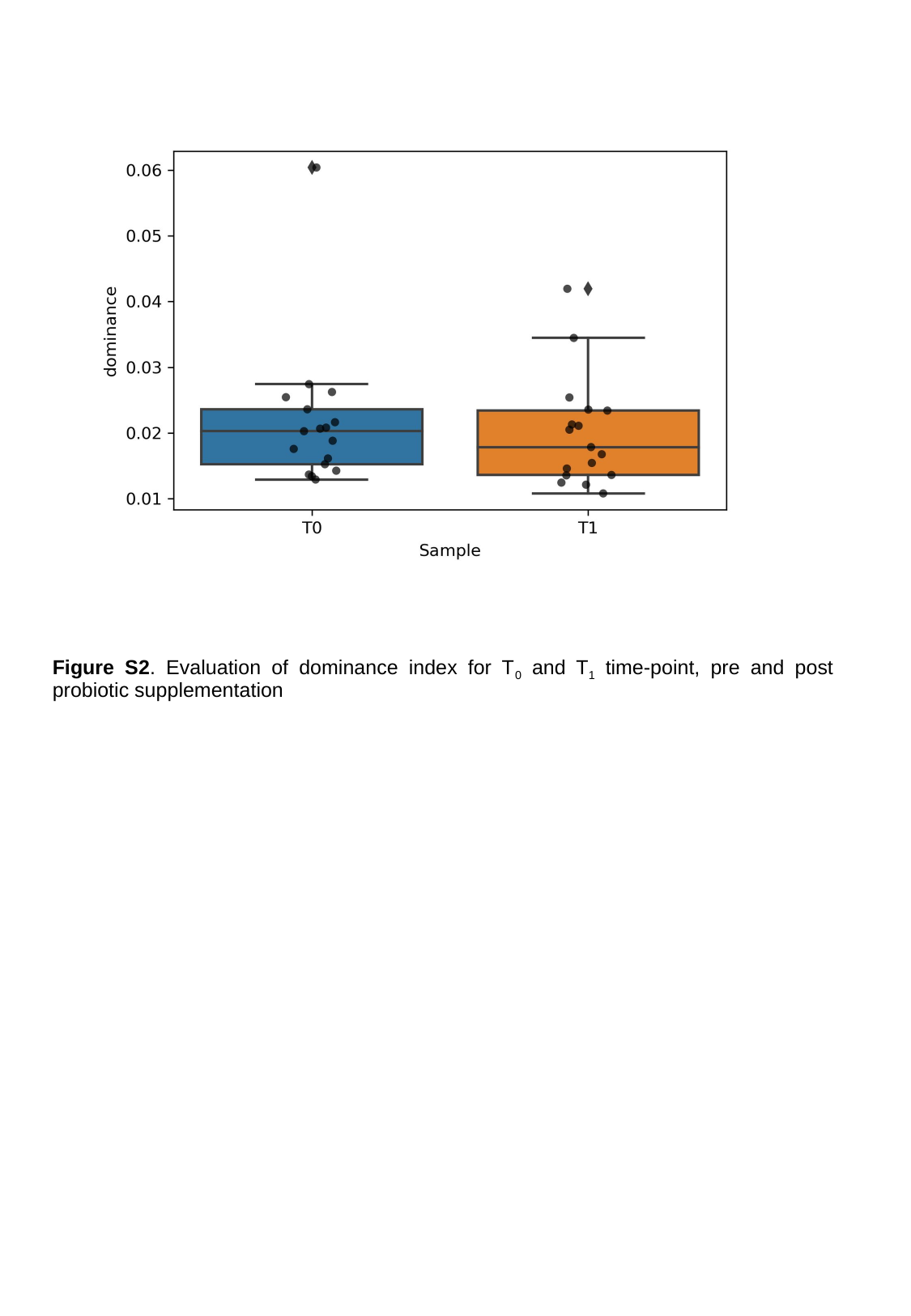

Figure S2. Evaluation of dominance index for T0 and T1 time-point, pre and post probiotic supplementation

## Slide 3
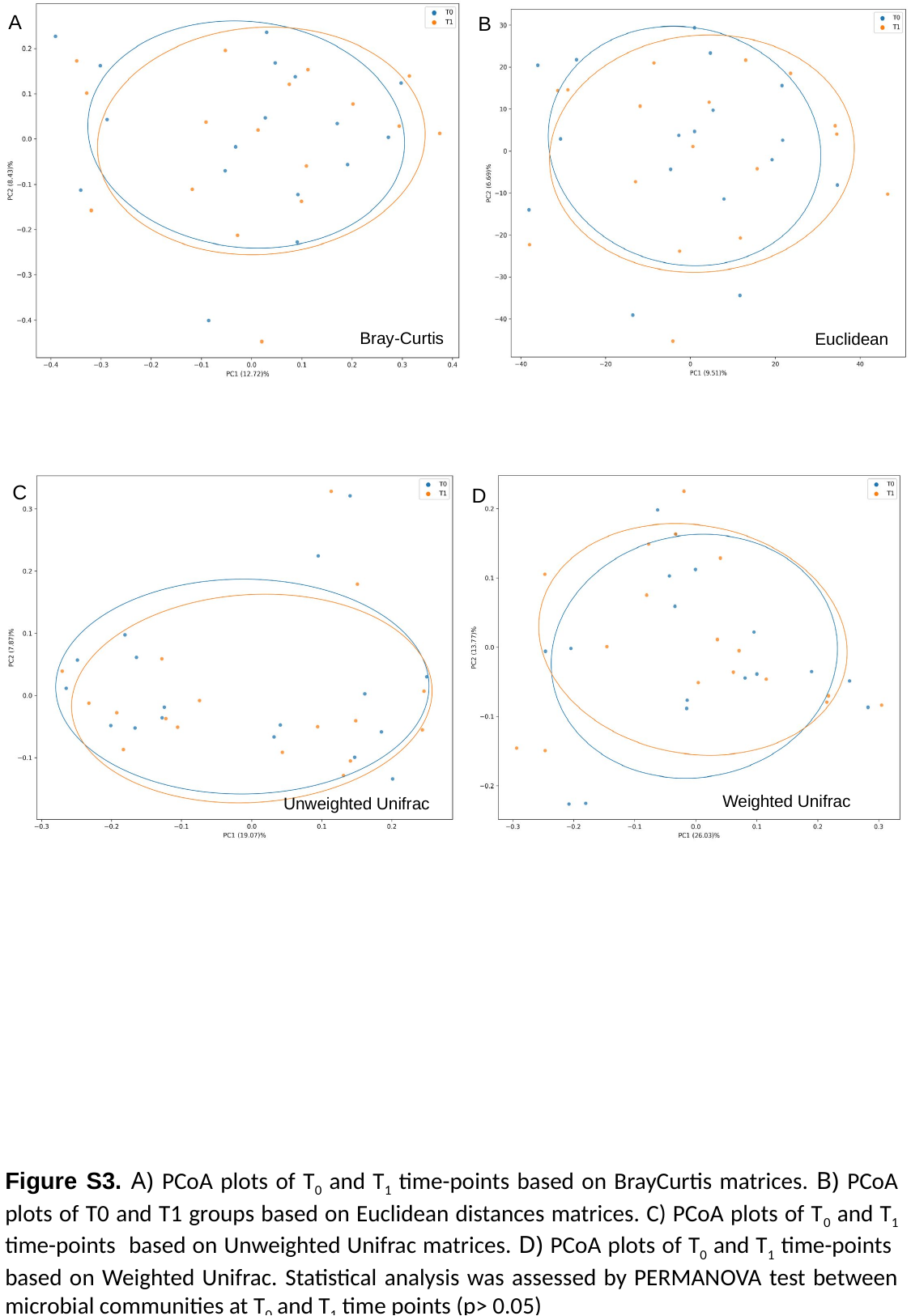

A
B
Bray-Curtis
Euclidean
C
D
Weighted Unifrac
Unweighted Unifrac
Figure S3. A) PCoA plots of T0 and T1 time-points based on BrayCurtis matrices. B) PCoA plots of T0 and T1 groups based on Euclidean distances matrices. C) PCoA plots of T0 and T1 time-points based on Unweighted Unifrac matrices. D) PCoA plots of T0 and T1 time-points based on Weighted Unifrac. Statistical analysis was assessed by PERMANOVA test between microbial communities at T0 and T1 time points (p> 0.05)

## Slide 4
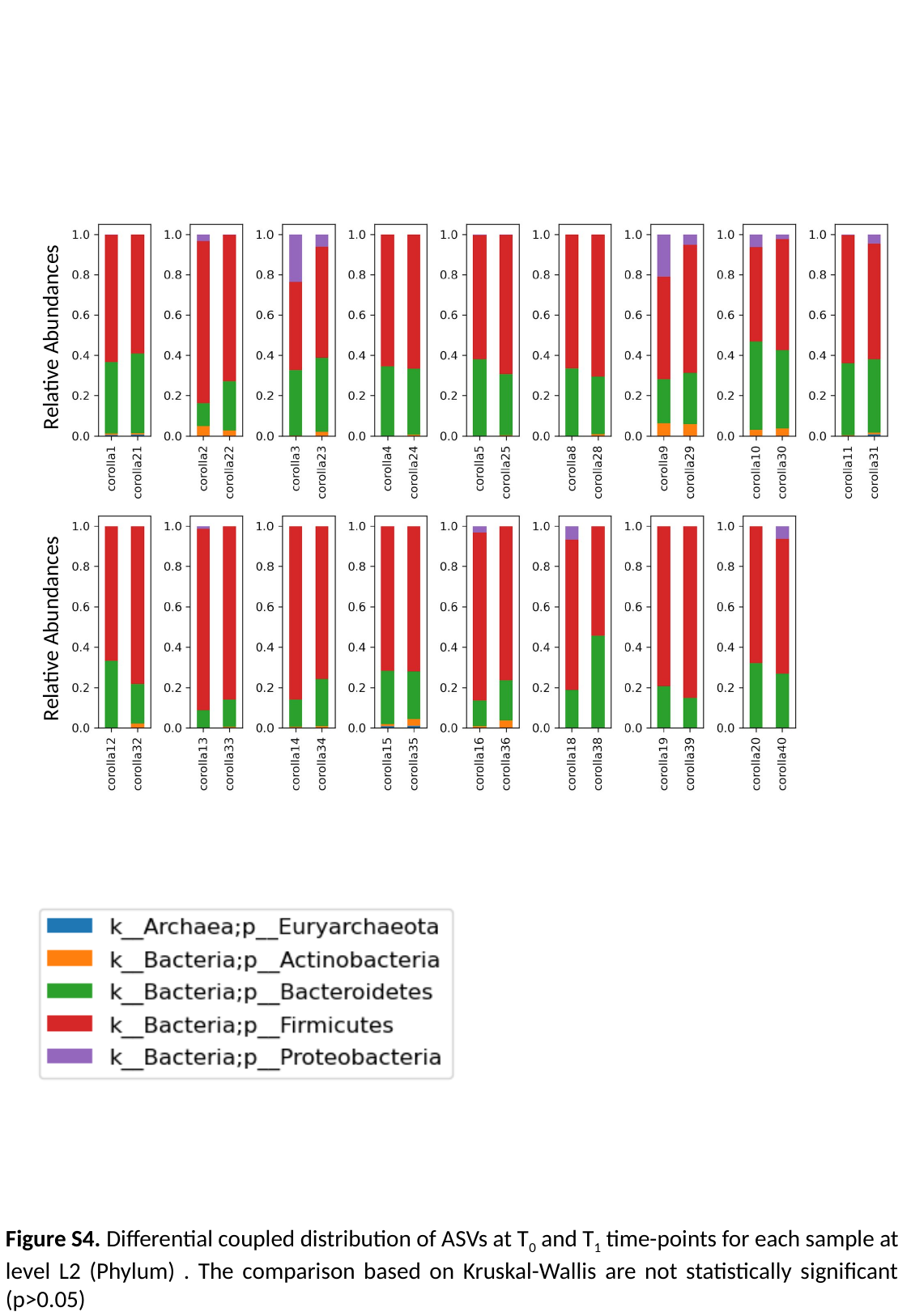

Relative Abundances
Relative Abundances
Figure S4. Differential coupled distribution of ASVs at T0 and T1 time-points for each sample at level L2 (Phylum) . The comparison based on Kruskal-Wallis are not statistically significant (p>0.05)

## Slide 5
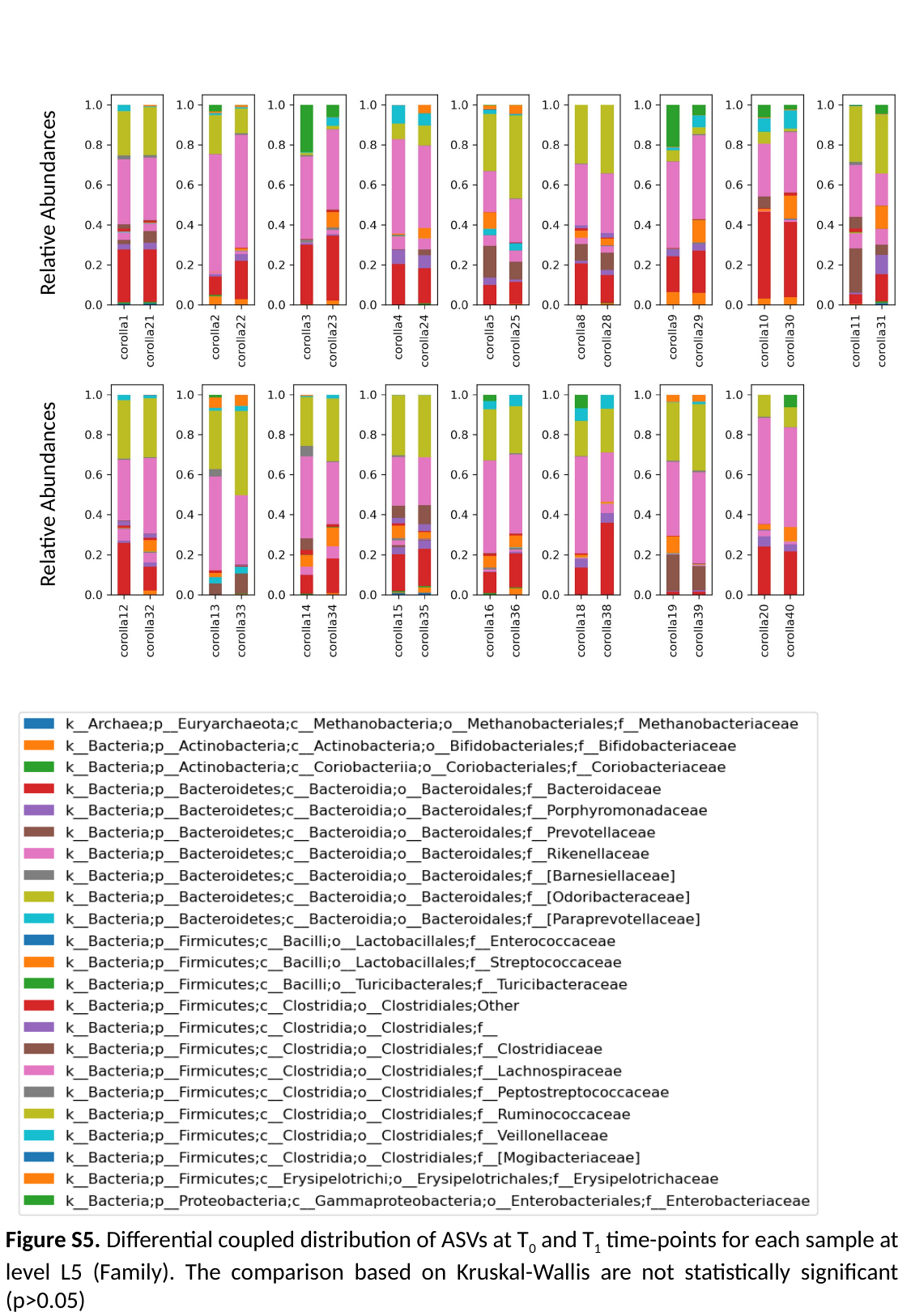

Relative Abundances
Relative Abundances
Figure S5. Differential coupled distribution of ASVs at T0 and T1 time-points for each sample at level L5 (Family). The comparison based on Kruskal-Wallis are not statistically significant (p>0.05)

## Slide 6
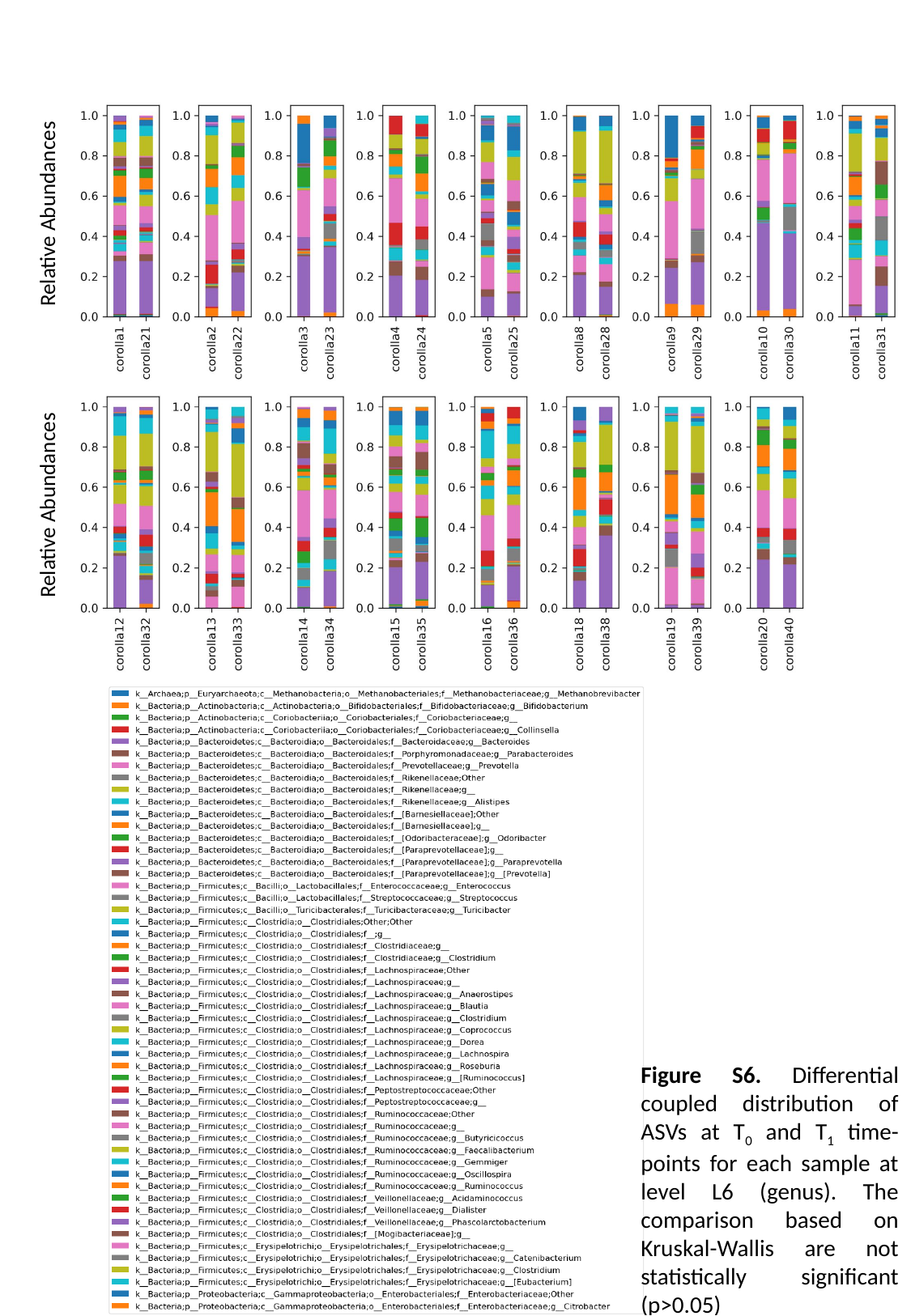

Relative Abundances
Relative Abundances
Figure S6. Differential coupled distribution of ASVs at T0 and T1 time-points for each sample at level L6 (genus). The comparison based on Kruskal-Wallis are not statistically significant (p>0.05)

## Slide 7
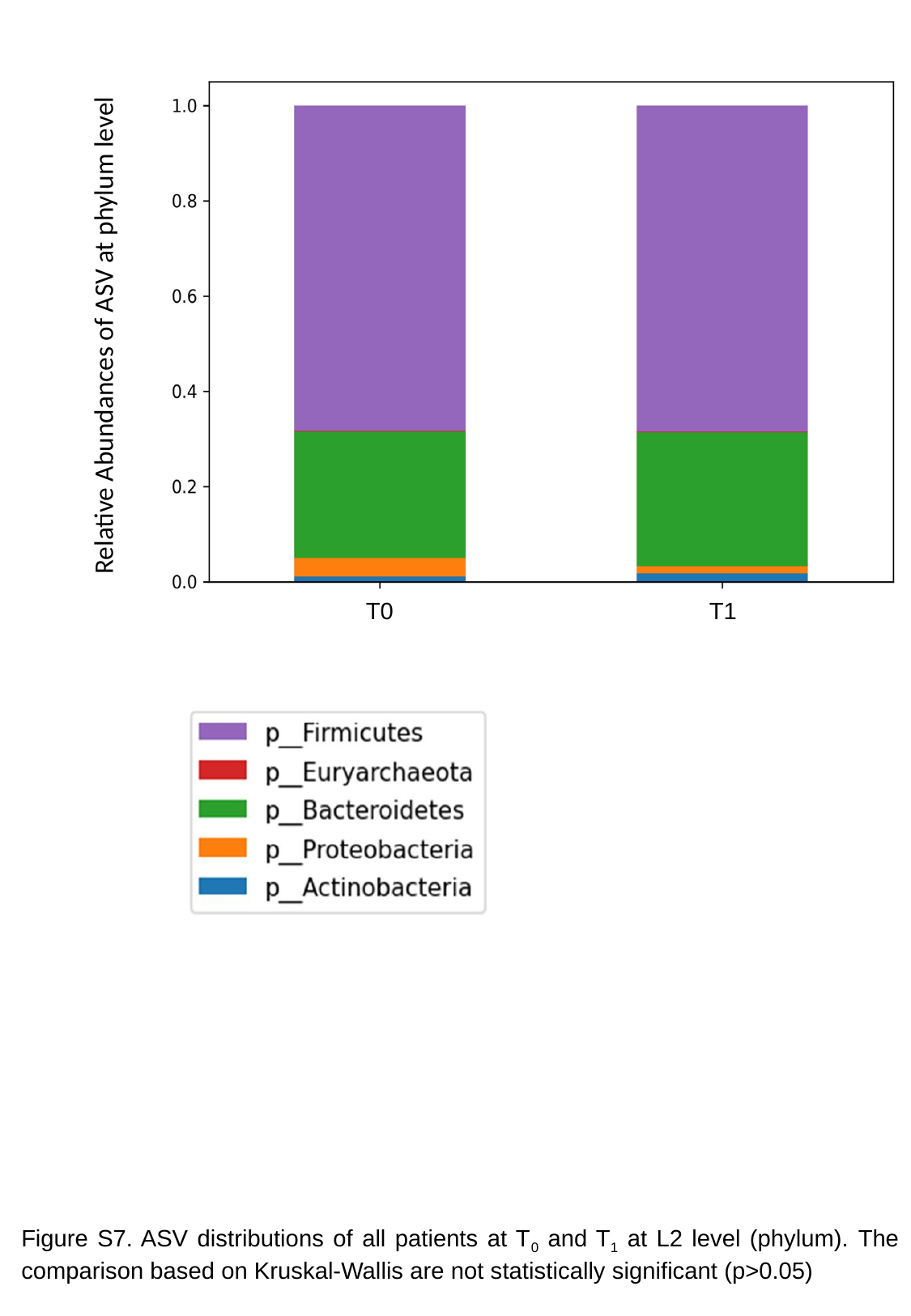

Relative Abundances of ASV at phylum level
T0
T1
Figure S7. ASV distributions of all patients at T0 and T1 at L2 level (phylum). The comparison based on Kruskal-Wallis are not statistically significant (p>0.05)
