# Supplementary material for: Ecology and Machine Learning-Based Classification Models of Gut Microbiota and Inflammatory Markers May Evaluate the Effects of Probiotic Supplementation in Patients Recently Recovered from COVID-19
Source: Int J Mol Sci. 2023 Apr 1;24(7):6623. doi: 10.3390/ijms24076623 (PMC10094838; doi:10.3390/ijms24076623)
Supplement: Supplementary file 1 [file ijms-24-06623-s001.zip › Table S1- GSRS global and single items scores.pdf]

Supplementary Table S1. GSRS global and single items scores.

		Patients (n=19*)			
	Summary statistics	Baseline (N=19)	Visit 3 (N=19)	Variation (Visit 3-Baseline)	P value
	N	11 (100.0%)	11 (100.0%)	11 (68.8%)	0.901
	Mean ± SD	19.55 ± 5.502	19.36 ± 5.390	-0.18 ± 4.750	
**Variation of GSRS**	Median (Q1-Q3)	17.00 (15.00-23.00)	18.00 (15.00-21.00)	0.00 (-4.00-2.00)	
	Min-Max	15.0-31.0	15.0-31.0	-9.0-7.0	
	Missing	0	0	5	
	N	14 (100.0%)	14 (100.0%)	14 (77.8%)	1.000
	Mean ± SD	1.79 ± 1.051	1.86 ± 1.562	0.07 ± 1.385	
**Abdominal pains**	Median (Q1-Q3)	2.00 (1.00-2.00)	1.00 (1.00-2.00)	0.00 (-1.00-0.00)	
	Min-Max	1.0-5.0	1.0-5.0	-1.0-4.0	
	Missing	0	0	4	
	N	14 (100.0%)	14 (100.0%)	14 (77.8%)	1.000
	Mean ± SD	1.07 ± 0.267	1.21 ± 0.802	0.14 ± 0.864	
**Heartburn**	Median (Q1-Q3)	1.00 (1.00-1.00)	1.00 (1.00-1.00)	0.00 (0.00-0.00)	
	Min-Max	1.0-2.0	1.0-4.0	-1.0-3.0	
	Missing	0	0	4	
	N	14 (100.0%)	14 (100.0%)	14 (77.8%)	1.000
	Mean ± SD	1.36 ± 0.842	1.21 ± 0.426	-0.14 ± 0.535	
**Acid Reflux**	Median (Q1-Q3)	1.00 (1.00-1.00)	1.00 (1.00-1.00)	0.00 (0.00-0.00)	
	Min-Max	1.0-4.0	1.0-2.0	-2.0-0.0	
	Missing	0	0	4	
	N	14 (100.0%)	14 (100.0%)	14 (77.8%)	0.313
	Mean ± SD	1.93 ± 0.829	1.64 ± 0.745	-0.29 ± 0.726	
**Hunger Pains**	Median (Q1-Q3)	2.00 (1.00-3.00)	1.50 (1.00-2.00)	0.00 (-1.00-0.00)	
	Min-Max	1.0-3.0	1.0-3.0	-2.0-1.0	
	Missing	0	0	4	
	N	14 (100.0%)	14 (100.0%)	14 (77.8%)	1.000
	Mean ± SD	1.07 ± 0.267	1.07 ± 0.267	0.00 ± 0.392	
**Nausea**	Median (Q1-Q3)	1.00 (1.00-1.00)	1.00 (1.00-1.00)	0.00 (0.00-0.00)	
	Min-Max	1.0-2.0	1.0-2.0	-1.0-1.0	
	Missing	0	0	4	
	N	12 (100.0%)	12 (100.0%)	12 (70.6%)	1.000
	Mean ± SD	1.42 ± 0.515	1.33 ± 0.492	-0.08 ± 0.515	
**Rumbling**	Median (Q1-Q3)	1.00 (1.00-2.00)	1.00 (1.00-2.00)	0.00 (0.00-0.00)	
	Min-Max	1.0-2.0	1.0-2.0	-1.0-1.0	
	Missing	0	0	5	
**Bloated**	N	12 (100.0%)	12 (100.0%)	12 (70.6%)	1.000

Patients (n=19*)

	Summary statistics	Baseline (N=19)	Visit 3 (N=19)	Variation (Visit 3-Baseline)	P value
	Mean ± SD	1.58 ± 0.996	1.58 ± 0.996	0.00 ± 0.603	
	Median (Q1-Q3)	1.00 (1.00-2.00)	1.00 (1.00-2.00)	0.00 (0.00-0.00)	
	Min-Max	1.0-4.0	1.0-4.0	-1.0-1.0	
	Missing	0	0	5	
**Burping**	N	12 (100.0%)	12 (100.0%)	12 (70.6%)	1.000
	Mean ± SD	1.17 ± 0.389	1.17 ± 0.389	0.00 ± 0.603	
	Median (Q1-Q3)	1.00 (1.00-1.00)	1.00 (1.00-1.00)	0.00 (0.00-0.00)	
	Min-Max	1.0-2.0	1.0-2.0	-1.0-1.0	
	Missing	0	0	5	
**Passing gas or flatus**	N	13 (100.0%)	13 (100.0%)	13 (76.5%)	0.258
	Mean ± SD	2.08 ± 1.115	1.69 ± 0.630	-0.38 ± 1.044	
	Median (Q1-Q3)	2.00 (1.00-3.00)	2.00 (1.00-2.00)	0.00 (-1.00-0.00)	
	Min-Max	1.0-4.0	1.0-3.0	-2.0-1.0	
	Missing	0	0	4	
**Constipation**	N	13 (100.0%)	13 (100.0%)	13 (76.5%)	0.750
	Mean ± SD	1.62 ± 1.193	1.38 ± 0.870	-0.23 ± 0.927	
	Median (Q1-Q3)	1.00 (1.00-1.00)	1.00 (1.00-1.00)	0.00 (0.00-0.00)	
	Min-Max	1.0-4.0	1.0-4.0	-3.0-1.0	
	Missing	0	0	4	
**Diarrhea**	N	13 (100.0%)	13 (100.0%)	13 (76.5%)	1.000
	Mean ± SD	1.08 ± 0.277	1.00 ± 0.000	-0.08 ± 0.277	
	Median (Q1-Q3)	1.00 (1.00-1.00)	1.00 (1.00-1.00)	0.00 (0.00-0.00)	
	Min-Max	1.0-2.0	1.0-1.0	-1.0-0.0	
	Missing	0	0	4	
**Loose stools**	N	13 (100.0%)	13 (100.0%)	13 (76.5%)	1.000
	Mean ± SD	1.08 ± 0.277	1.00 ± 0.000	-0.08 ± 0.277	
	Median (Q1-Q3)	1.00 (1.00-1.00)	1.00 (1.00-1.00)	0.00 (0.00-0.00)	
	Min-Max	1.0-2.0	1.0-1.0	-1.0-0.0	
	Missing	0	0	4	
**Hard stools**	N	13 (100.0%)	13 (100.0%)	13 (76.5%)	0.375
	Mean ± SD	1.77 ± 1.363	1.31 ± 0.480	-0.46 ± 1.330	
	Median (Q1-Q3)	1.00 (1.00-2.00)	1.00 (1.00-2.00)	0.00 (-1.00-0.00)	
	Min-Max	1.0-5.0	1.0-2.0	-4.0-1.0	
	Missing	0	0	4	
**Urgent need to have a bowel movement**	N	13 (100.0%)	13 (100.0%)	13 (76.5%)	1.000
	Mean ± SD	1.38 ± 0.870	1.38 ± 0.870	0.00 ± 0.913	
	Median (Q1-Q3)	1.00 (1.00-1.00)	1.00 (1.00-1.00)	0.00 (0.00-0.00)	

Patients (n=19*)

	Summary statistics	Baseline (N=19)	Visit 3 (N=19)	Variation (Visit 3-Baseline)	P value
	Min-Max	1.0-4.0	1.0-4.0	-2.0-2.0	
	Missing	0	0	4	
**Sensation of not completely emptying the bowels**	N	12 (100.0%)	12 (100.0%)	12 (75.0%)	1.000
	Mean ± SD	1.33 ± 0.651	1.33 ± 0.888	0.00 ± 0.603	
	Median (Q1-Q3)	1.00 (1.00-1.50)	1.00 (1.00-1.00)	0.00 (0.00-0.00)	
	Min-Max	1.0-3.0	1.0-4.0	-1.0-1.0	
	Missing	0	0	4	

*The number refers to all patients (19) that have completed the protocol
