# Supplementary material for: Ecology and Machine Learning-Based Classification Models of Gut Microbiota and Inflammatory Markers May Evaluate the Effects of Probiotic Supplementation in Patients Recently Recovered from COVID-19
Source: Int J Mol Sci. 2023 Apr 1;24(7):6623. doi: 10.3390/ijms24076623 (PMC10094838; doi:10.3390/ijms24076623)
Supplement: Supplementary file 1 [file ijms-24-06623-s001.zip › Table S2-Frequency distribution of individual items for GSRS score.pdf]

Supplementary Table S2. Frequency distribution of individual items for GSRS score.

		Patients (n=19*)		
		Summary statistics	Baseline	Visit 3
**1. Abdominal pains**	No discomfort	%, n	42.9% (6/14)	71.4% (10/14)
	Minor discomfort	%, n	50.0% (7/14)	7.1% (1/14)
	Moderate discomfort	%, n		7.1% (1/14)
	Severe discomfort	%, n	7.1% (1/14)	14.3% (2/14)
**2. Heartburn**	No discomfort	%, n	92.9% (13/14)	92.9% (13/14)
	Minor discomfort	%, n	7.1% (1/14)	
	Moderate discomfort	%, n		7.1% (1/14)
**3. Acid Reflux**	No discomfort	%, n	78.6% (11/14)	78.6% (11/14)
	Minor discomfort	%, n	14.3% (2/14)	21.4% (3/14)
	Moderate discomfort	%, n	7.1% (1/14)	
**4. Hunger Pains**	No discomfort	%, n	35.7% (5/14)	50.0% (7/14)
	Minor discomfort	%, n	35.7% (5/14)	35.7% (5/14)
	Moderate discomfort	%, n	28.6% (4/14)	14.3% (2/14)
**5. Nausea**	No discomfort	%, n	92.9% (13/14)	92.9% (13/14)
	Minor discomfort	%, n	7.1% (1/14)	7.1% (1/14)
**6. Rumbling**	No discomfort	%, n	58.3% (7/12)	66.7% (8/12)
	Minor discomfort	%, n	41.7% (5/12)	33.3% (4/12)
**7. Bloated**	No discomfort	%, n	66.7% (8/12)	66.7% (8/12)
	Minor discomfort	%, n	16.7% (2/12)	16.7% (2/12)
	Mild discomfort	%, n	8.3% (1/12)	8.3% (1/12)
	Moderate discomfort	%, n	8.3% (1/12)	8.3% (1/12)
**8. Burping**	No discomfort	%, n	83.3% (10/12)	83.3% (10/12)
	Minor discomfort	%, n	16.7% (2/12)	16.7% (2/12)
**9. Passing gas or flatus**	No discomfort	%, n	38.5% (5/13)	38.5% (5/13)
	Minor discomfort	%, n	30.8% (4/13)	53.8% (7/13)
	Mild discomfort	%, n	15.4% (2/13)	7.7% (1/13)
	Moderate discomfort	%, n	15.4% (2/13)	
**10. Constipation**	No discomfort	%, n	76.9% (10/13)	76.9% (10/13)
	Minor discomfort	%, n		15.4% (2/13)
	Mild discomfort	%, n	7.7% (1/13)	
	Moderate discomfort	%, n	15.4% (2/13)	7.7% (1/13)
**11. Diarrhea**	No discomfort	%, n	92.3% (12/13)	100.0% (13/13)
	Minor discomfort	%, n	7.7% (1/13)	
**12. Loose stools**	No discomfort	%, n	92.3% (12/13)	100.0% (13/13)
	Minor discomfort	%, n	7.7% (1/13)	

Patients (n=19*)

		Summary statistics	Baseline	Visit 3
**13. Hard stools**	No discomfort	%, n	69.2% (9/13)	69.2% (9/13)
	Minor discomfort	%, n	7.7% (1/13)	30.8% (4/13)
	Mild discomfort	%, n	7.7% (1/13)	
	Moderate discomfort	%, n	7.7% (1/13)	
	Severe discomfort	%, n	7.7% (1/13)	
**14. Urgent need to have a bowel movement**	No discomfort	%, n	76.9% (10/13)	76.9% (10/13)
	Minor discomfort	%, n	15.4% (2/13)	15.4% (2/13)
	Moderate discomfort	%, n	7.7% (1/13)	7.7% (1/13)
**15. Sensation of not completely emptying the bowels**	No discomfort	%, n	75.0% (9/12)	83.3% (10/12)
	Minor discomfort	%, n	16.7% (2/12)	8.3% (1/12)
	Mild discomfort	%, n	8.3% (1/12)	
	Moderate discomfort	%, n		8.3% (1/12)

*The number refers to all patients (19) that have completed the protocol
